# Analgesic and Anti-Inflammatory Properties of Extracts from the Bulbils of *Dioscorea bulbifera* L. var *sativa* (Dioscoreaceae) in Mice and Rats

**DOI:** 10.1155/2011/912935

**Published:** 2010-09-08

**Authors:** M. Mbiantcha, A. Kamanyi, R. B. Teponno, A. L. Tapondjou, P. Watcho, T. B. Nguelefack

**Affiliations:** ^1^Laboratory of Animal Physiology and Phytopharmacology, Faculty of Science, University of Dschang, P.O. Box 67, Dschang, Cameroon; ^2^Laboratory of Environmental and Applied Chemistry, Faculty of Science, University of Dschang, P.O. Box 183, Dschang, Cameroon

## Abstract

The aqueous and methanol extracts from the dry bulbils of *Dioscorea bulbifera* L. var sativa (Dioscoreaceae)—evaluated orally at the doses of 300 and 600 mg/kg against pain induced by acetic acid, formalin, pressure and against inflammation induced by carrageenan, histamine, serotonin and formalin in mice and rats, showed a dose dependant inhibition of pain and inflammation with a maximum effect of 56.38%, 73.06% and 42.79% produced by the aqueous extract, respectively on pain induced by acetic acid, formalin and pressure while the methanol extract at the same dose respectively inhibited these models of pain by 62.70%, 84.54% and 47.70%. The oral administration of aqueous and methanol extracts caused significant anti-inflammatory activity on paw oedema induced by histamine, serotonin and formalin. The present results show that the bulbils of *Dioscorea bulbifera* var sativa possess potent analgesic and anti-inflammatory activities. These activities may results from the inhibition of inflammatory mediators such as histamine, serotonin and prostaglandins. Thus, the analgesic activity of the bulbils of *Dioscorea bulbifera* may be at least partially linked to its anti-inflammatory activity.

## 1. Introduction

Dioscoreaceae are distributed throughout the tropics and constitute a family consisting mainly of tropical climbers. Almost all species are perennial herbaceous or shrubby climbers with well-developed tubers or rhizomes. Leaves are usually alternate, simple, but sometimes palmately lobed or compound [1]. *Dioscorea* is a genus in the monocotyledonous family Dioscoreaceae [2]. *Dioscorea bulbifera* L. is a glabrous nonspiny climber of 10–20 feet high with bulbils 1–8 cm in size [3]. Tubers are toxic or edible according to the variety; they are renewed annually. *Dioscorea bulbifera* L. var *sativa* commonly called “letoucfong” in the Bamboutos division (West Province of Cameroon) grows wild and has bitter tubers and bulbils [4]. A steroidal saponin, spiroconazole A, a phenanthrene, 2,7-dihydroxy-4-methoxyphenanthrene, flavonoids as quercetin, quercetin-3-O-*β*-D-glucopyranoside, and quercetin-3-O-*β*-D-galactopyranoside, and seven clerodane diterpenoids namely, bafoudiosbulbins A, B, C, D, E, F, and G have been isolated from the methanol extract of the bulb of *Dioscorea bulbifera* var sativa [5–8]. *Dioscorea bulbifera* L. var *sativa* is used in Bangladesh for the treatment of leprosy and tumours [4] and by the native people of the western highlands of Cameroon for the treatment of pig Cysticercosis, though the tubers, after collection during the farming period, are totally destroyed and burnt because of their high bitterness. The roots of *Dioscorea bulbifera* L., although considered poisonous because of cytotoxic activity, have been used in Chinese medicine as a remedy for sore throat and for struma [9]. In Zimbabwe, this plant is used as an infusion to apply on cuts and sores, both for humans and animals while in Cameroon and Madagascar, the pounded bulbs are applied to abscesses, boils, and wound infections [10]. Its bulbs are used in India to treat piles, dysentery, syphilis and are applied to ulcers, pain, and inflammation [11]. Although the plant material has been long used to treat pain and inflammation, no scientific work has been carried out to ascertain the claimed properties. The present work was then undertaken to evaluate the analgesic and anti-inflammatory effects of the aqueous and methanol extracts from the bulbils of *Dioscorea bulbifera* in mice and rats.

## 2. Materials and Methods

### 2.1. Plant Material

The bulbils of *Dioscorea bulbifera* L. var *sativa* were collected in Bafou village near Dschang (West province of Cameroon) in February 2007. The plant was identified by Dr G. Achoundong, Cameroon National Herbarium, Yaoundé, where a voucher specimen (Ref: 22211/SRF/CAM) was deposited.

The aqueous extract of the bulbils of *Dioscorea bulbifera* was obtained by maceration of 5 kg of the dried and pulverized bulbils in distilled water for 3 days. The filtrate was evaporated at 40°C to produce 156 g of a solid residue, corresponding to a yield of 3.12%.

The dried and pulverized bulbils of *Dioscorea bulbifera* (12 kg) were extracted two times (each time for 24 hours) with MeOH-H_2_O (8 : 2). The filtrate obtained was concentrated under reduced pressure to yield 530 g of a dark residue, representing the methanol extract (4.42% yield).

For the phytochemical screening, the test of Shinoda was used to reveal the presence of flavonoids [12], Ehrich's test for furanoids [13], while saponins were revealed as described by Hostettmann et al. [14].

### 2.2. Animals

White adult mice *Mus musculus* (weighing 25–30 g) and adult Wistar rats (weighing 180–200 g), of either sex, obtained from the animal house of the Laboratory of Animal Physiology and Phytopharmacology of the University of Dschang (Cameroon) were used in the present study. These animals were raised under natural conditions (temperature: 21 ± 2°C; 12 h/12 h light-dark cycle) and were allowed free access to water and food. Experiments were carried out on groups of 6 animals each. All experimental procedures used in the present study followed the “Principles of Laboratory Animal Care” from NIH publication Nos. 85-23 and were approved by the ethic committee of the Cameroon Ministry of Scientific Research and Technology which has adopted the guidelines established by the European Union on Animal Care and Experimentation (CEE Council 86/609).

### 2.3. Chemicals

Acetic acid, formaldehyde, pyrilamine maleate, carrageenan, histamine, serotonin, DMSO, and tween 80 were obtained from Sigma Chemical Co. (St.Louis, MO, USA), while indomethacin (Indocid, Merck Sharp-Dolme Chibret, Fr.), aspirin (UPSA), and diclofenac (Olfen-100 SR) were obtained from a local pharmacy. All these chemicals and the aqueous extract were prepared in normal saline (0.9%) except indomethacin that was dissolved in 3% DMSO. The methanol extract was prepared in a mixture of 2.5% DMSO and 2.5% tween 80.

### 2.4. Analgesic Activity 

#### 2.4.1. Animal Allotment and Treatment

In each analgesic test, seven groups of animals were used. Groups 1 and 2 served as negative controls and were treated with distilled water and mixture of Tween (2.5%)/DMSO (2.5%), respectively. Group 3 was used as positive control and received indomethacin (10 mg/kg). The 4 remaining groups were treated with the aqueous or methanol extract of the bulbils of *Dioscorea bulbifera *at the doses of 300 and 600 mg/kg. All the treatments were administered orally.

#### 2.4.2. Acetic Acid-Induced Abdominal Writhing Test

This test was performed as previously described by H. G. Vogel and W. H. Vogel [15]. One hour after administration of the treatments, acetic acid solution (0.6%; v/v) was injected intraperitoneally (0.1 mL/10 g) to each animal. The number of writhing produced in these animals was counted for 30 min, starting 5 min after acid injection. The percentage of the analgesic activity was calculated as follows:

(1) $$\text{Inhibition}\left(\%\right)=\frac{\bar{N}c-\bar{N}t}{\bar{N}c}\times 100,$$

where $\bar{N}$ *c* = mean number of writhing in control group; $\bar{N}$ *t* = mean number of writhing in treated group.

#### 2.4.3. Formalin Test

The formalin-induced paw licking was studied in mice using the method described by Nguelefack et al. [16]. In this method, 20 *μ*L of 2.5% formalin was injected into the subcutaneous tissue of the plantar surface of the left hind paw of rats 1 hour after administration of drugs. The time the animal spent licking the injected paw was counted in two different phases: from 0–5 min postinjection (early phase) and from 15–30 min postinjection (late phase). The percentage of analgesic activity at each phase was calculated using the following formula:

(2) $$\text{Inhibition}\left(\%\right)=\frac{\bar{C}-\bar{T}}{\bar{C}}\times 100,$$

with $\bar{C}$ = mean time in control group for each phase and $\bar{T}$ = mean time in treated group for each phase.

#### 2.4.4. Analgesy Meter Test

This experiment was conducted as previously described by Kamanyi et al. [17]. The rat was suspended vertically while its left hind paw placed between the plinth and the finger of the Ugo Basile analgesy meter. This instrument generates a linearly increasing mechanical force or pressure by dome-shaped on the dorsal surface on the rat's hind paw. As the applied pressure increases, it gets to a point where the animal struggles to free its paw. This point was taken as the threshold level at which the animal feels pain. This threshold point was determined before, 0.5, 1, 2, and 4 h after treatment. Protection rates were calculated as follows:

(3) $$\text{Protection}\left(\%\right)=\frac{\left(Pt-Po\right)\text{test}-\left(Pt-Po\right)\text{control}}{\left(Pt-Po\right)\text{control}}\times 100,$$

with *P* *o* = threshold value before administration of drugs and *P* *t* = threshold value at time *t* after administration of drugs.

### 2.5. Anti-Inflammatory Activity 

#### 2.5.1. Carrageenan-Induced Paw Oedema

The experiment was carried out as described by Winter et al. [18]. Adult rats were divided and treated as described in the analgesic section. The paw volume of the rats was measured plesthysmographically (UGO BASILE-7140) before treatments (V0). One hour after treatment, 0.1 mL of carrageenan (1% in 0.9% NaCl) was administered into the subcutaneous tissue of the plantar surface of the right hind paw. Then the volume of paw was taken at 0.5, 1, 2, 3, 4, 5, and 6 hours after carrageenan administration (*V* *t*). The oedema was expressed as an increase in the volume of paw (Δ*V*), and the percentage of inhibition (*I*%) for each treatment was obtained as follows: Δ*V* = *V* *t* − *V*0

(4) $$I\left(\text{\%}\right)=\frac{\Delta Vc-\Delta V\mathrm{tr}\;}{\Delta Vc}\times 100,$$

where Δ*V*tr  = right hind paw average increased volume in treated group and Δ*V* *c* = right hind paw average increase in control groups.

#### 2.5.2. Paw Oedema Induced by Histamine and Serotonin

Animals were divided as in the previous experiment but the control group received pyrilamine maleate at 1 mg/kg. Oedema was induced by subcutaneous injection of 0.1 mL of freshly prepared solutions of serotonin (10 mg/mL) or histamine (1 mg/mL) into the hind paws of the rats 1 hour after oral administration of drugs. The volume of the injected paws was measured 30 min and 1 hour after injection of serotonin or histamine, respectively [19]. The percentage inhibition induced by each drug was calculated as in the carrageenan test.

#### 2.5.3. Formalin-Induced Paw Oedema

This experiment was undertaken using the methodology previously described by Dimo et al. [20]. Seven groups of rats were used: two negative controls treated with distilled water or with the mixture Tween (2.5%)/DMSO (2.5%), one positive control treated with diclofenac (10 mg/kg), and 4 groups receiving the aqueous or the methanol extract at the doses of 300 and 600 mg/kg. Thirty minutes later, 0.1 mL formalin (2% in 0.9% NaCl) was injected into the subplanter area of right hind paw of rats. The paw volume was determined acutely by the plethysmographic method before drugs' administration and 1, 2, and 4 hours following the injection of formalin. The same animals were used for the chronic test. They were treated daily after the measurement of the paw volume for 10 consecutive days. A second injection of formalin was given on the third day.

### 2.6. Statistical Analysis

Results are presented as mean ± Standard Error of the Mean (SEM). One-way ANOVA followed by the Tukey post test or two-ways ANOVA followed by Bonferroni post test were used where necessary for statistical analysis.

## 3. Results

### 3.1. Phytochemical Studies

Phytochemical screening of aqueous and methanol extracts revealed the presence of flavonoids, furanoids, and saponins.

### 3.2. Acetic Acid-Induced Writhing Test

**Figure 1:**
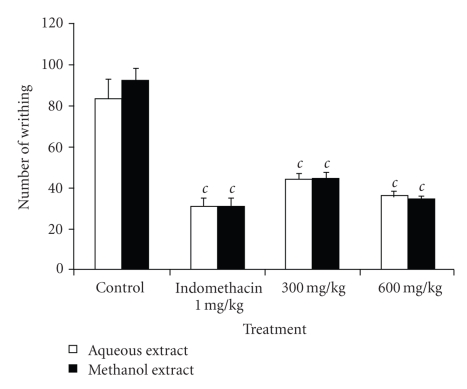
Effects of aqueous and methanol extracts of the bulbils of *Dioscorea bulbifera* L. var *sativa* on acetic acid-induced pain in mice. Each bar represents the mean ± SEM of 6 animals; ^*c*^ *P* < .001 statistically significant compared to their respective control.

The oral administration of the aqueous and methanol extracts of the bulbils of *Dioscorea bulbifera* in mice significantly (*P* < .001) reduced in a dose-dependent manner the number of mouse abdominal constrictions induced by acetic acid. The aqueous extract at doses of 300 and 600 mg/kg showed a protective effect of 47.02% and 56.38%, respectively. At the same doses, the methanol extract induced a protective effect of 51.89% and 62.70%, respectively. The methanol extract at 600 mg/kg exhibited the same activity as indomethacin (62.75%) (Figure 1).

### 3.3. Formalin Test

**Figure 2:**
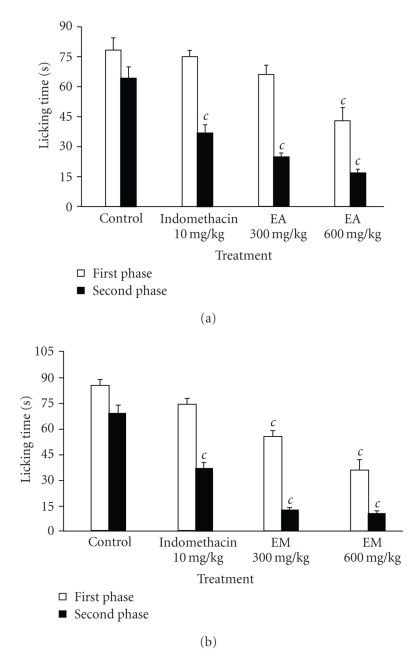
Effects of aqueous (EA) (a) and methanol (EM) (b) extracts of the bulbils of *Dioscorea bulbifera* L. var *sativa* on formalin-induced pain in mice. Each bar represents the mean ± SEM of 6 animals; ^*c*^ *P* < .001 statistically significant compared to their respective control.

The intraplantar injection of 20 *μ*L of formalin (2.5%) into the right hind paw generated a classical biphasic nociceptive response. As shown in Figure 2, plant extracts (300 and 600 mg/kg) significantly and dose-dependently reduced the nociception in both the early and late phases. The aqueous and methanol extract significantly reduced the licking time at the first phase of observation by 44.80% and 57.78% at 600 mg/kg, respectively. Indomethacin did not show significant activity on this first phase. During the second phase of observation, the aqueous and methanol extracts at the dose of 600 mg/kg showed a maximum protective effect of 73.06% and 84.54%, respectively while indomethacin significantly inhibited it by 42.22%.

### 3.4. Analgesy Meter Test

**Figure 3:**
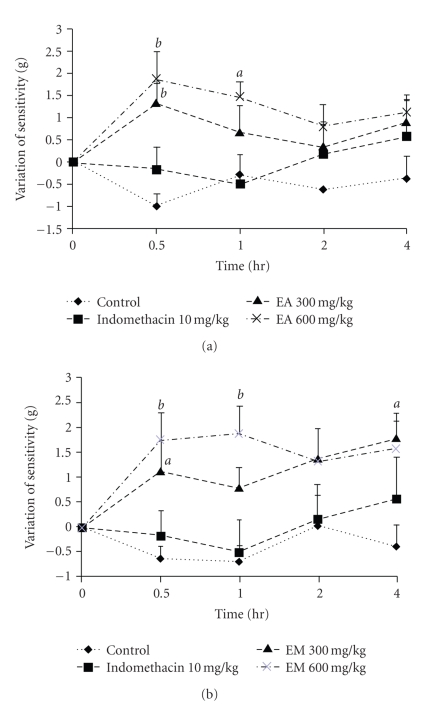
Antinociceptive effects of aqueous (EA) (a) and methanol (EM) (b) extracts of the bulbils of *Dioscorea bulbifera* L. var *sativa* in pressure-caused pain. Each point represents the mean ± SEM of 6 animals; ^*a*^ *P* < .05; ^*b*^ *P* < .01, statistically significant compared to their respective control.

The aqueous extract at the doses 300 and 600 mg/kg significantly (*P* < .01) reduced the animal sensitivity to pain induced by pressure, raising the threshold of reaction by 29.04% and 42.79%, respectively. The methanol extract induced an inhibition of 47.70% at the dose of 600 mg/kg while indomethacin did not show any significant effect (Figure 3).

### 3.5. Carrageenan-Induced Paw Oedema

**Figure 4:**
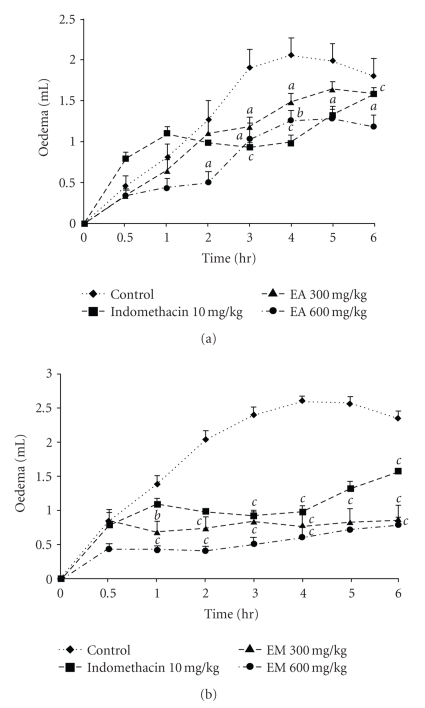
Anti-inflammatory effects of aqueous (EA) (a) and methanol (EM) (b) extracts of the bulbils of *Dioscorea bulbifera* L. var *sativa* on rat hind paw oedema induced by carrageenan. Each point represents the mean ± SEM of 6 animals; ^*a*^ *P* < .05; ^*b*^ *P* < .01, ^*c*^ *P* < .001 statistically significant compared to their respective control.

As it can be seen from Figure 4, the subplantar injection of the carrageenan produced an inflammatory oedema which increased gradually, reaching its maximum at the 4th hour after injection. The aqueous extract at the dose 600 mg/kg exhibited an anti-inflammatory activity that became significant 2 hours after the injection of carrageenan and was maintained all along the experiment with a maximum of 60.63%. The methanol extract (300 and 600 mg/kg) induced significant (*P* < .001) anti-inflammatory effect that increased progressively and reached a maximum (80%) at the second hour. Indomethacin (10 mg/kg) exhibited an anti-inflammatory effect with a maximum response of 62.36% (*P* < .001) at the fourth hour.

### 3.6. Histamine and Serotonin Induced Paw Oedema

**Figure 5:**
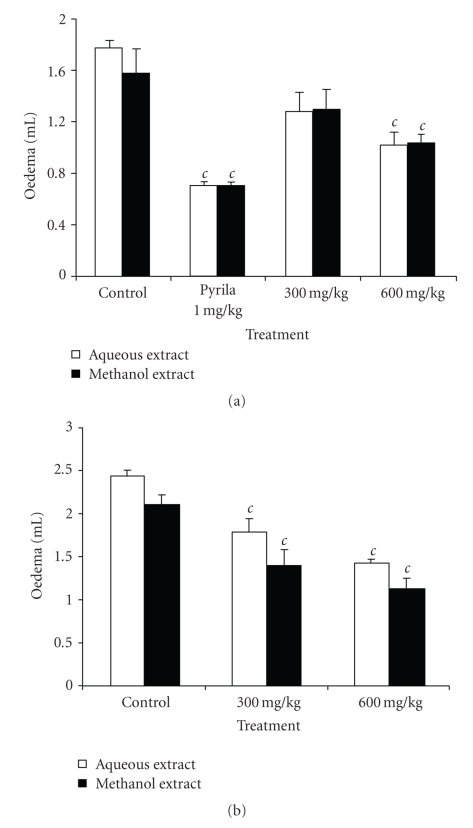
Effects of aqueous and methanol extracts of the bulbils of *Dioscorea bulbifera* L. var *sativa* on paw oedema induced by histamine (a) and serotonin (b). Each bar represents the mean ± SEM of 6 animals; ^*c*^ *P* < .001 statistically significant compared to their respective control.

Figure 5 shows the effects of aqueous and methanol extracts on paw oedema induced by histamine and serotonin. Both extracts exhibited a significant dose-dependent anti-inflammatory activity with a maximum of 42.37% and 34.39% in histamine-induced oedema and 42.04% and 46.92% in serotonin-induced oedema, respectively for the aqueous and methanol extracts at the dose of 600 mg/kg. Pyrilamine maleate significantly (*P* < .001) inhibited by 59.89% the inflammation induced by histamine.

### 3.7. Formalin-Induced Paw Oedema

**Figure 6:**
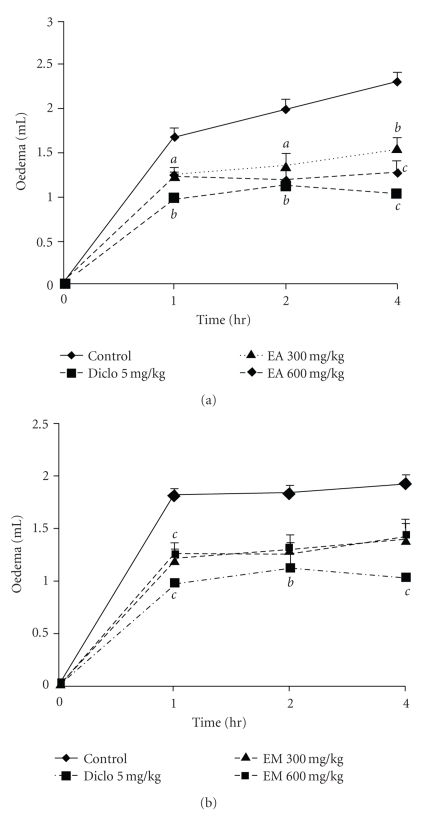
Effects of aqueous (EA) (a) and methanol (EM) (b) extracts of the bulbils of *Dioscorea bulbifera* L. var *sativa* on acute inflammation of paw oedema induced by formalin. Each point represents the mean ± SEM of 6 animals; ^*b*^ *P* < .01; ^*c*^ *P* < .001 statistically significant compared to their respective control.

The aqueous and methanol extract of the bulbils of *Dioscorea bulbifera* significantly and dose dependently inhibited inflammation induced by formalin. At the dose of 600 mg/kg, they significantly reduced the formation of paw oedema by 45.02% for the aqueous extract and 32.06% for the methanol extract, respectively at the 2nd and the 4th hours. Diclofenac significantly inhibited the acute inflammation induced by formalin by 55.41% at the 4th hour (Figure 6).

**Figure 7:**
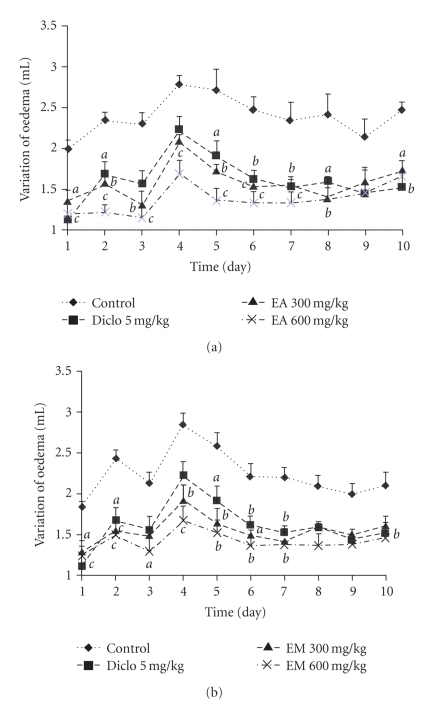
Effects of aqueous (EA) (a) and methanol (EM) (b) extracts of the bulbils of *Dioscorea bulbifera* L. var *sativa* on chronic inflammation of paw oedema induced by formalin. Each point represents the mean ± SEM of 6 animals; ^*a*^ *P* < .05; ^*b*^ *P* < .01, ^*c*^ *P* < .001 statistically significant compared to negative control.

Both extracts showed a significant inhibition of chronic inflammation. The aqueous extract (300 and 600 mg/kg) significantly inhibited the formaldehyde arthritis by 43.48% and 50.43%, respectively on day 3. At the same doses, methanol extract inhibited the chronic inflammation by 36.43% and 40.84%, respectively on days 4 and 5 (Figure 7).

## 4. Discussion

Results from the present study showed that the aqueous and methanol extracts from the bulbils of *Dioscorea bulbifera var Sativa *have a potent antinociceptive effect against chemical pains provoked by acetic acid or formalin and a slight activity against mechanic pain induced by pressure. These extracts also presented important anti-inflammatory effects on acute oedema induced by carrageenan, histamine, serotonin, or formalin, and on chronic oedema induced by formalin.

The acetic acid-induced abdominal constriction method is widely used for the evaluation of peripheral antinociceptive activity [21] because it is very sensitive and able to detect antinociceptive effects of compounds at dose levels that may appear inactive in other methods [22, 23]. Local peritoneal receptors are postulated to be partly involved in the abdominal constriction response [24]. The method has also been associated with prostanoids in general, for example, increased levels of PGE_2_ and PGF_2*α*_ in peritoneal fluids [25] as well as lipoxygenase products [26, 27]. The aqueous and methanol extracts of *D. bulbifera* as indomethacin exhibited marked inhibitory effect on the writhing response induced by acetic acid. These results strongly suggest that these extracts possess peripheral analgesic activity and their mechanisms of action may be mediated through inhibition of local peritoneal receptors or arachidonic acid pathways, involving cyclo-oxygenases and/or lipoxygenases. The significant analgesic effects observed on analgesy meter test buttress the effect of the extracts on peripheral local receptors even though the extracts were less effective on this model.

In the formalin test, there is a distinctive biphasic nociceptive response termed early and late phases. Drugs that act primarily on the central nervous system inhibit both phases equally while peripherally acting drugs inhibit the late phase [28, 29]. The early phase is probably a direct result of stimulation of nociceptors in the paw and reflects centrally mediated pain while the late phase is due to inflammation with a release of serotonin, histamine, bradykinin, and prostaglandins [30] and at least to some degree, the sensitization of central nociceptive neurons [30–32]. Suppression of both phases of pain was observed in animals treated with aqueous and methanol extracts. These results lend strong credence to the presence of both central and peripheral effects. Since *D. bulbifera* extracts were very efficient on visceral pain induced by acetic acid and on the late phase of pain induced by formalin all mediated by histamine, serotonin, bradykinin, and prostaglandins, it is possible that these extracts possess anti-inflammatory activities. 

In order to verify this hypothesis, the aqueous and the methanol extracts were assayed on acute and chronic inflammation models. The paw oedema induced by carrageenan involves several chemical mediators such as histamine, serotonin, bradykinin, and prostaglandins [33, 34]. In the carrageenin-induced rat paw oedema model, *Dioscorea bulbifera *extracts showed significant inhibitory effect on the oedema formation. This effect started from the first hour and was maintained in all the inflammatory phases, suggesting that the main mechanism of action of the tested extracts may involve prostaglandin biosynthesis pathway and may influence other mediators of inflammation. To ascertain the effect of the aqueous and methanol extracts on the mediators' activities, they were tested on inflammation induced by histamine and serotonin. It was observed that the plant extracts were capable of inhibiting oedema induced by histamine and serotonin. Furthermore, oedema induced by formalin was also significantly inhibited by both extracts. Acute inflammation induced by formalin results from cell damage, which provokes the production of endogenous mediators, such as, histamine, serotonin, prostaglandins, and bradykinin [35]. It can then be concluded from these results that *D. bulbifera *extracts interfere with these inflammatory mediators. But was it possible that these interesting acute anti-inflammatory effects extended to chronic inflammations which are the most invaliding factors? 

To answer this question, both extracts were tested against formalin-induced oedema which is a typical feature of an established chronic inflammatory reaction and can serve as a subchronic and chronic inflammatory test model for investigation of antiarthritic and probable antiproliferative substances [36]. The aqueous and methanol extracts showed significant inhibitory effects on this model, suggesting that the plant extracts possess subchronic anti-inflammatory activities and may also serve as antiarthritic and antiproliferative medicine. Phytochemical screening revealed the presence of flavonoids, furanoids, and saponins in the bulbils of *Dioscorea bulbifera*. Several flavonoids with significant antinociceptive and/or anti-inflammatory effects have been isolated from medicinal plants [37–40]. Moreover, quercetin and its derivatives that possess analgesic activities have been isolated from the methanol extract of the bulbils of *Dioscorea bulbifera *[5–8]. It is therefore, possible that both the antinociceptive and anti-inflammatory effects observed with these extracts are attributed to its flavonoid components.

**Figure 8:**
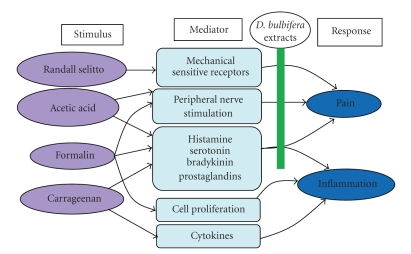
Probable mechanism of the analgesic and anti-inflammatory activities of *Dioscorea bulbifera* extracts.

In conclusion, this study has shown that the aqueous and methanolic extracts of the bulbils of *Dioscorea bulbifera* L. var *sativa* do possess significant antinociceptive and anti-inflammatory effects that may be mediated through inhibition of cell mediators such as histamine, serotonin, bradykinin, and prostaglandins (Figure 8). These results support the traditional use of this plant in some painful and inflammatory conditions.
